# Comparison of the prognostic ability of quantitative traumatic methods patients admitted to ‎Imam Khomeini Hospital of Urmia in 2016

**Published:** 2019-08

**Authors:** Omid Garkaz, Shaker Salari Lak, Hamid Reza Mehryar, Hamid Reza Khalkhali

**Affiliations:** ^*a*^Urmia University of Medical Sciences, Urmia, Iran.; ^*b*^Department of Public Health, Islamic Azad University, Tabriz Branch, Tabriz, Iran.

## Abstract

**Background:**

Trauma is the major cause of disability in industrial societies and its management needs major ‎asset in order to setting up specialized trauma centers. In Iran trauma is the main cause of long ‎term capability. The aim of this study is to compare the prognostic ability of the several ‎traumatic measurements which conventionally were used. Patients which refered to Urmia ‎Imam Khomeini University Hospital in 2016 enrolled in our study. ‎

**Methods:**

In this study severity of trauma in patients with traffic accident referred to Urmia Imam ‎Khomeini University hospital determined using ISS, RTS.TRISS, and ASCOT scoring systems ‎along with one-year period. Demographic data, mortality rate, hospitalization days and total ‎hospital costs were collected and analyzed by regression methods and ROC curve. For this ‎purpose, the records of hospital casualties were reviewed. ‎

**Results:**

The mean age of the patients was 33.63 ± 18.53 and the age range was between 1-96 years. The ‎ratio of male to female was 2.73. The average severity of lesions based on the ISS system was ‎‎16.44 ± 16.28 and 16.8% of the ISS injured were over 25. The difference in severity of trauma ‎‎(calculated by each of the methods (ISS, RTS, TRISS, ASCOT) were significant between ‎survivors and those who died. The ISS system had the highest correlation with hospital stay ‎‎(0.616) and hospital costs (0/123). There was a significant correlation between the four methods ‎with hospitalization and hospitalization days, but the coefficient of determination in each of the ‎four methods was from 0/167 (length of hospitalization) and hospital cost (0/123) did not go. ‎

**Conclusions:**

In this study, the highest mortality rate was related to the RTS and TRISS methods and the best ‎way to predict hospital costs and duration of hospitalization was to use the ISS method. ‎Considering the usefulness and special applications of these methods.‎

**Keywords:**

Trauma, Accident, TRISS, ISS, RTS, ASCOT

